# Differential Globalization of Industry- and Non-Industry–Sponsored Clinical Trials

**DOI:** 10.1371/journal.pone.0145122

**Published:** 2015-12-14

**Authors:** Ignacio Atal, Ludovic Trinquart, Raphaël Porcher, Philippe Ravaud

**Affiliations:** 1 Centre d’Épidémiologie Clinique, Hôpital Hôtel-Dieu, Paris, France; 2 INSERM U1153, Paris, France; 3 Columbia University, Mailman School of Public Health, Epidemiology Department, New York, New York, United States of America; 4 Université Paris Descartes, Paris, France; York University, CANADA

## Abstract

**Background:**

Mapping the international landscape of clinical trials may inform global health research governance, but no large-scale data are available. Industry or non-industry sponsorship may have a major influence in this mapping. We aimed to map the global landscape of industry- and non-industry–sponsored clinical trials and its evolution over time.

**Methods:**

We analyzed clinical trials initiated between 2006 and 2013 and registered in the WHO International Clinical Trials Registry Platform (ICTRP). We mapped single-country and international trials by World Bank's income groups and by sponsorship (industry- vs. non- industry), including its evolution over time from 2006 to 2012. We identified clusters of countries that collaborated significantly more than expected in industry- and non-industry–sponsored international trials.

**Results:**

119,679 clinical trials conducted in 177 countries were analysed. The median number of trials per million inhabitants in high-income countries was 100 times that in low-income countries (116.0 vs. 1.1). Industry sponsors were involved in three times more trials per million inhabitants than non-industry sponsors in high-income countries (75.0 vs. 24.5) and in ten times fewer trials in low- income countries (0.08 vs. 1.08). Among industry- and non-industry–sponsored trials, 30.3% and 3.2% were international, respectively. In the industry-sponsored network of collaboration, Eastern European and South American countries collaborated more than expected; in the non-industry–sponsored network, collaboration among Scandinavian countries was overrepresented. Industry-sponsored international trials became more inter-continental with time between 2006 and 2012 (from 54.8% to 67.3%) as compared with non-industry–sponsored trials (from 42.4% to 37.2%).

**Conclusions:**

Based on trials registered in the WHO ICTRP we documented a substantial gap between the globalization of industry- and non-industry–sponsored clinical research. Only 3% of academic trials but 30% of industry trials are international. The latter appeared to be conducted in preferentially selected countries.

## Introduction

Clinical trials are fundamental in advancing knowledge and improving health care globally.[1,2] By evaluating health interventions, clinical trials bring evidence about pharmacological and non-pharmacological therapies. International collaboration in clinical trials offers numerous advantages for the generation and interpretation of evidence.[3–6] Apart from accelerating the accrual of patients, especially for uncommon diseases, an important advantage in operating trials across countries is to increase the applicability of research findings. International collaboration in health research may also play an important role in reducing waste in health research.[7] Moreover, international clinical trials may strengthen health care systems in emerging economies because externally sponsored trials may increase the research capacity of sites in developing countries.[2,3]

As recently stated in *Science*, "the issue of knowing what research is currently being undertaken—where, by whom, and which organizations are supporting it—is a black hole in the public health landscape".[8] The international landscape of health research should be mapped to inform global governance and policy development.[9] In the last two decades, the number of clinical trials has expanded worldwide, and developing countries are increasingly involved, with a migration of trials from North America and Europe to Asia and Latin America.[10–12] Unravelling the forces that shape the research agenda may help steer it toward the most relevant health issues, to address the disparity between the local health burden and the production of health knowledge through clinical trials.[13,14]

A specific area of concern is the extent to which the clinical research landscape is dominated by industry sponsors.[15] In particular, international collaboration in clinical trials is constrained by scientific, ethical, economical, operational, and regulatory considerations. Different sponsors may have different capacities to address these constraints, and industry- and non-industry–sponsored research may thus show different collaborative patterns. Recent work suggest that private biomedical R&D expenditures in the United States have been reallocated to Asia and Oceania in the last five years.[16] Indeed, the pharmaceutical industry may be increasingly using global networks.[12] To our best knowledge, no quantitative large-scale data on this issue are available.

In 2006, the World Health Organization (WHO) established the International Clinical Trials Registry Platform (ICTRP), which gathers 16 worldwide registries of clinical trials meeting criteria of content, accessibility, quality and validity.[17] Based on clinical trials registered included in the WHO ICTRP, we aimed to map the global landscape of industry- and non-industry–sponsored clinical trials and its evolution over time.

## Methods

To analyse the global landscape of clinical trials, we used data for all registered clinical trials that were included in the WHO ICTRP. We first mapped the trials and then studied the system of country–country collaboration for industry- and non-industry–sponsored clinical trials. Collaboration between two countries was defined as the number of international clinical trials conducted simultaneously in at least these two countries. We analysed the system of country-country collaboration by deriving networks of collaboration for industry- and non-industry–sponsored clinical trials. In these networks, each node represents a country and an edge between two countries represents the number of international trials conducted simultaneously in at least these two countries. To describe the patterns of collaborations, we analysed these networks with a complex systems approach as detailed below.

### Data

We retrieved records of clinical trials registered before February 2, 2014 in the ICTRP. After eliminating duplicates, we extracted the start date, the primary sponsor and the country locations for each trial (for details, see S1 Appendix). Because since September 2005, the International Committee of Medical Journal Editors has required registration before considering a trial for publication, we restricted the analysis to clinical trials with start dates between 2006 and 2013.[18]

Trials were classified by sponsor type (industry or non-industry) based on the primary sponsor, defined in the WHO ICTRP as the “organization which takes responsibility for the initiation, management, and/or financing of a clinical trial”. The sponsor type was available for trials registered in ClinicalTrials.gov (90.7% of all included trials). For each of the remaining trials (9.3%), we determined whether the primary sponsor name matched that of the trial registered in ClinicalTrials.gov. If no match was found, we used a pre-specified list of keywords such as "Ltd.", "Inc.", and "University" to categorize the primary sponsor (for a detailed list, see S1 Appendix). We excluded 2.5% of all trials for which the sponsor type remained unclear.

The geographic classification of countries was based on the GeoNames and EuroVoc databases as well as the Organization for Economic Co-operation and Development (OECD) classification.[19,20] The country populations and income classifications were obtained from the World Bank database 2012.

### Mapping of clinical trials

We first mapped the global distribution of clinical trials. Second, we mapped industry- and non- industry–sponsored clinical trials and the proportion of industry-sponsored trials. Finally, we mapped single-country and international clinical trials and the proportion of international trials for each sponsor type.

These mappings were performed both at the country-level and for groups of countries. At the country level, we mapped the density of clinical trials as the number of trials per million inhabitants. The density was considered only in countries with more than 250,000 inhabitants. At the country level, the share of sponsorship and of international trials was considered only in countries with at least more than 50 trials initiated (in total, industry- or non-industry–sponsored depending on the analysis) during the 2006–2013 period.

### Collaboration network analysis

We analysed the industry- and non-industry–sponsored networks of collaboration using a null model analysis and a cluster analysis. In a network of collaboration, each node represents a country and an edge between two countries represents the country-country collaboration, corresponding to the number of international trials conducted simultaneously in at least these two countries.

To asses if some country-country collaborations were overrepresented as compared to what would be expected because of chance, we conducted a so-called null-model analysis, as developed in the field of ecology.[21,22] For a given pair of countries, this method compares the observed number of collaborative trials between two given countries to the distribution of this number under the null hypothesis that all countries collaborate with each other purely at random. We derived the null distributions by generating 90,000 networks of collaboration through a permutation-based algorithm which preserved the numbers of trials initiated in each country and the numbers of countries involved in each trial (i.e., the margins of the collaboration matrix).[23] To test for overrepresentation, we compared each observed country-country collaboration to the 99.9th percentile of the corresponding null distribution. For each null distribution of country-country collaboration, 90,000 random networks of collaboration allowed for identifying the value below which 99.9% ± 0.01% of observations fall. To avoid sparse collaboration matrices, we had suppressed the countries participating in the lowest numbers of international trials. We removed countries successively until 95% and 90% of the total country-country collaborations remained for industry- and non-industry–sponsored networks, respectively.

For overrepresented country-country collaborations, we measured the extent to which both countries were involved more than expected by chance in the same international trials. This degree of overrepresentation was estimated as the ratio of the distance between the observed country-country collaboration and the mean of the null distribution to the distance between the 99.9th percentile and the mean of the null distribution. Then, we constructed co-occurrence networks where each node represents a country and an edge connects two countries if their collaboration is overrepresented, in which case its width corresponds to the degree of overrepresentation as previously.

To identify groups of countries where collaboration was higher than expected, we conducted a cluster analysis on the co-occurrence networks. Cluster analysis of networks is a data-driven approach allowing a network to be partitioned into groups to provide a simpler understanding of the network structure. The clustering algorithm we used partitioned the countries into clusters whereby the flow of collaboration is maximized within a cluster and minimized between clusters.[24] Countries in the same cluster were more likely to be involved together or with the same countries in clinical trials, and countries in different clusters had fewer chances of being involved together or with common countries in clinical trials.

### Evolution over time

We studied the evolution of the mappings over time. Because of retrospective registration of trials, which may be more prevalent for trials that started in 2013, we restricted the time evolution analysis to the 2006–2012 period.[25] We computed the mappings for each year of the period and checked if trends existed.

All analyses involved use of R 3.0.2,[26] except for cluster analysis, which involved InfoMap code 0.13.5,[27] and co-occurrence network visualization, which involved NodeXL 1.0.1.251.[28]

### Ethics statement

An ethics statement was not required for this work.

## Results

We analysed 119, 679 clinical trials initiated during the 2006–2013 period (S1 Dataset). These trials were conducted in 177 countries, accounting for 99.3% of the worldwide population. In all, 30.1% of trials were industry-sponsored and 69.9% non-industry–sponsored (S1 Fig).

### Global mapping of clinical trials

**Fig 1:**
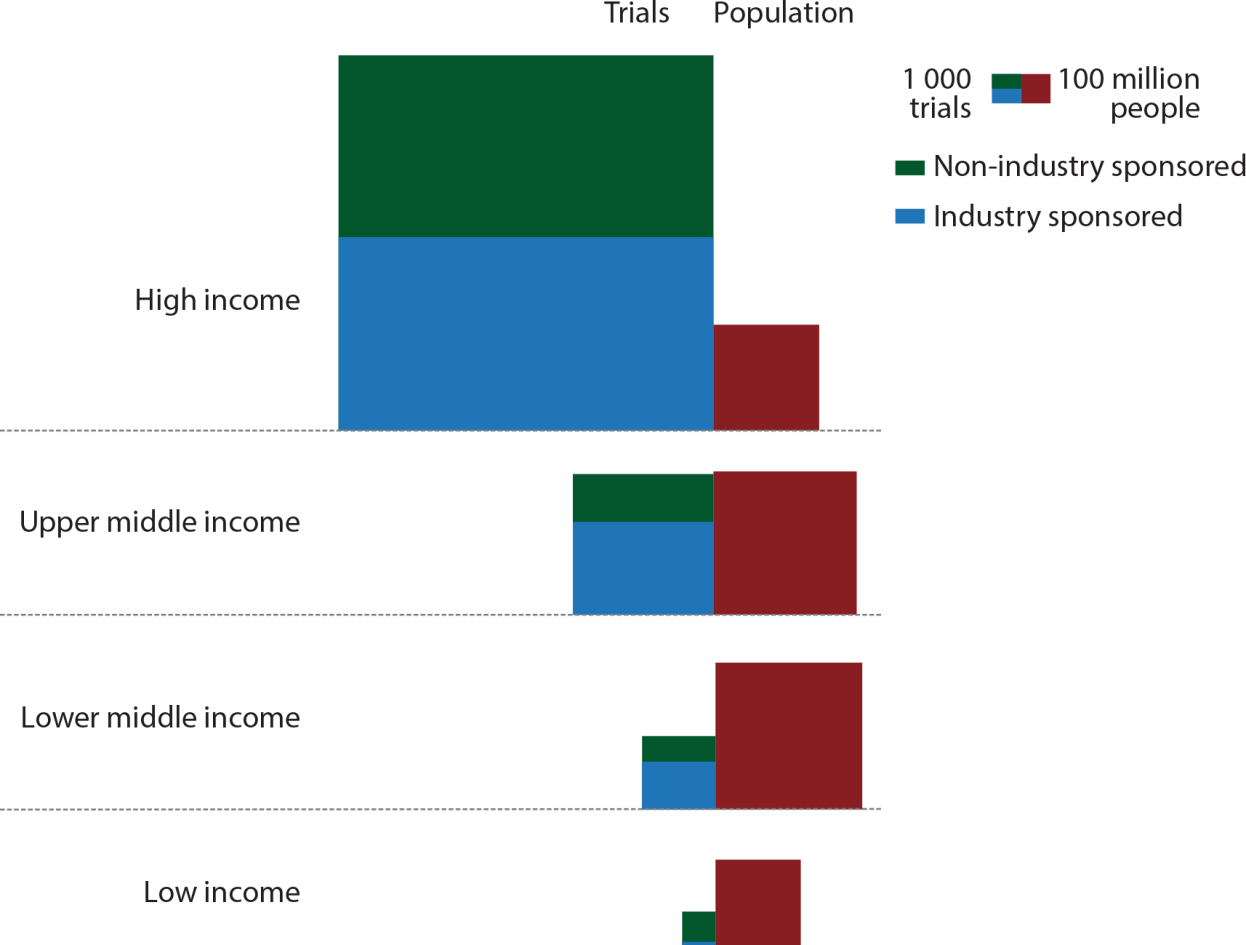
Distribution of clinical trials and population per income groups. For each income group, the size of the green (blue, respectively) area is proportional to the number of industry-sponsored (non-industry–sponsored) trials initiated during the 2006–2013 period, and the size of the red area is proportional to the population as of 2012. Equal-sized trial and population squares correspond to an overall density of 10 trials per million inhabitants. The proportion of industry-sponsored clinical trials was 51.6%, 66.0%, 65.4% and 9.3% in high-, upper-middle-, lower-middle- and low-income countries, respectively. In high-income countries, the density of trials ranged from 2.2 trials per million inhabitants in Trinidad and Tobago to 645.7 for Denmark. In upper-middle-income countries, it ranged from 0.05 to 225.9, with more than 50 trials per million inhabitants in four countries, all in Eastern Europe (Hungary, Bulgaria, Romania and Serbia). The variation was less pronounced in lower-middle-income countries (between 0.04 and 22.6) and low- income countries (between 0.13 and 14.0).

Overall, the number of trials conducted in each country was extremely variable between income groups (Fig 1). The median number of trials per million inhabitants was 116.0 in high-income countries, 13.8 in upper-middle-income countries, but 1.8 and 1.1 in lower-middle- and low-income countries, respectively. In all, 1.65 billion people (23.4% of the world population) lived in countries where less than two trials per million inhabitants were initiated, most in low- or lower-middle- income countries (92.2%). The regions with the highest median density of clinical trials were Western Europe and Eastern Europe with 166.59 and 76.24 trials per million inhabitants, respectively (S2 Fig and S1 Table).

### Sponsorship mapping

**Fig 2:**
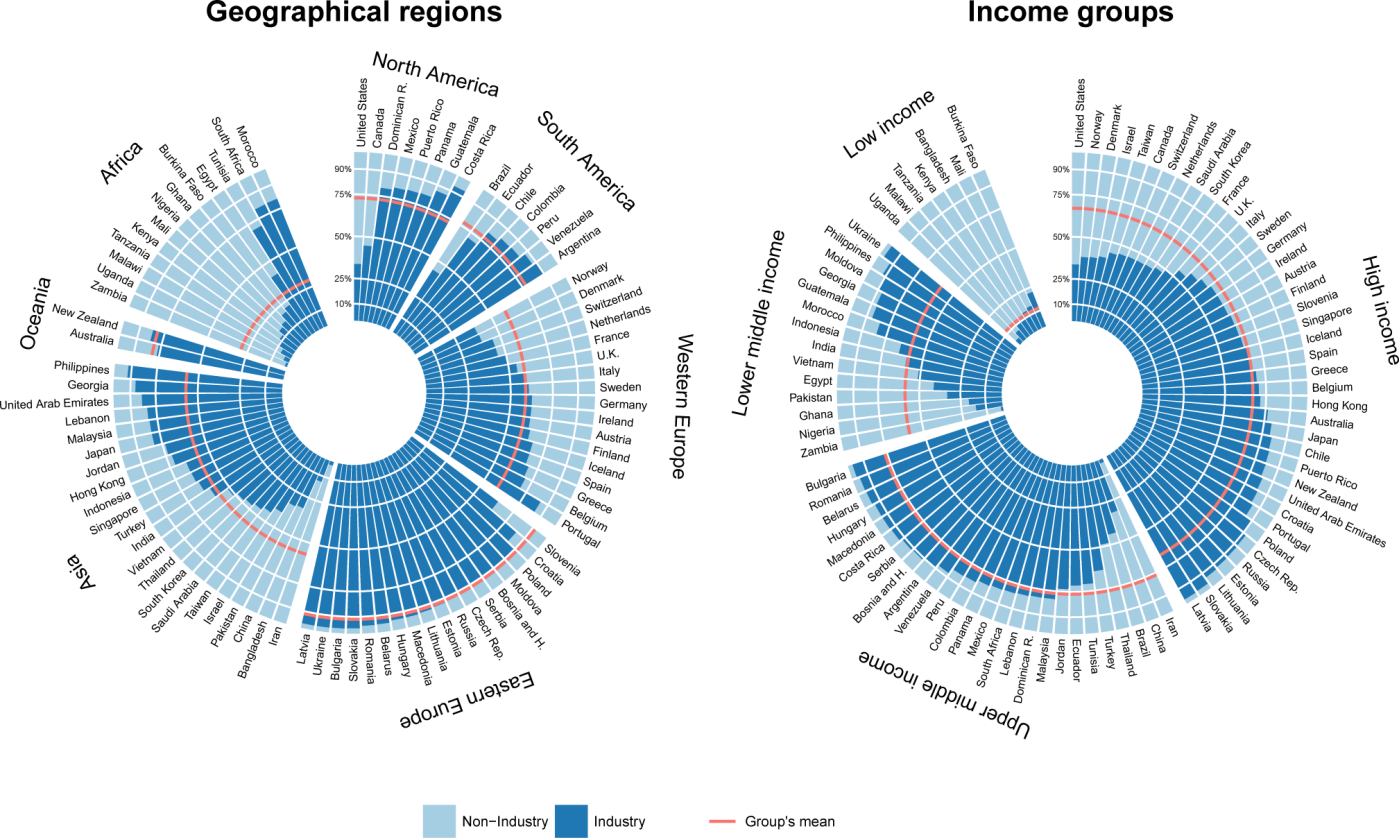
Sponsorship ratios of clinical trials. The radial barplot shows the proportion of industry- sponsored clinical trials (in dark blue) in the 87 countries where at least 50 trials were initiated during the 2006–2013 period. Countries were grouped by (a) geographical region and (b) income groups. For each group of countries, the red line represents the mean proportion of industry-sponsored clinical trials. The exact sponsorship ratios per country can be found on S4 Table. In high-income countries the proportion of industry-sponsored trials ranged from 33.6% in the United States to 90% or more in seven Eastern European countries. In upper-middle- income countries, the proportion ranged from 2.1% for Iran to more than 97% for countries such as Bulgaria and Romania. In lower-middle-income countries, the proportion ranged from less than 20% in three African countries to 97.2% in Ukraine.

The proportion of industry-sponsored trials showed substantial variations across geographical regions and income groups. In particular, the proportion of industry-sponsored trials was 91.5% in Eastern Europe, 58.9% in Western Europe and 29.2% in Africa (Fig 2A). Similarly, the proportion of industry-sponsored trials was 67.0% and 76.4% in high- and upper-middle-income countries, as compared to 11.7% in low-income countries (Fig 2B). In all income groups except the low-income group, the proportion of industry-sponsored trials varied, with the highest proportion consistently in Eastern European countries. In low-income countries, the proportion of industry-sponsored trials was homogeneously low as compared to the other income groups.

In high- and upper-middle-income countries, the median number of industry-sponsored trials per million inhabitants was three times that of non-industry–sponsored trials (75.0 vs. 24.5 and 7.3 vs. 2.5, respectively). In lower-middle-income countries, the three-fold difference was reversed (0.23 vs. 0.90), and in low-income countries the median number of industry- sponsored trials per million inhabitants was ten times less that of non-industry–sponsored trials (0.08 vs. 1.08). In fact, in all low-income countries but one, less than one industry-sponsored clinical trial per million inhabitants was initiated between 2006 and 2013.

### Collaboration mapping

#### Global collaboration mapping

Most trials were conducted in a single country (88.6%). Single-country trials were mainly conducted in high-income countries (88.6%), particularly in the United States (42.3%), Western Europe (30.6%), and Asia (16.6%). Among international trials, 43.5% were conducted in a single continent. International single-continental trials were mainly conducted in Europe (65.8%) and North America (25.1%). Moreover, more than 90% of all international trials were conducted in at least one North American or Western European country, and United-States–Canada collaborations represented 25.8% of international trials (3,523 trials).

#### Differences between industry- and non-industry–sponsored collaborations

Most of the single-country trials were non-industry sponsored (76.3%). The distribution of single- country trials by income groups and geographical regions was similar for both sponsor types (S3 and S5 Figs.). Most of the international trials were industry-sponsored (80.1%). The proportion of international trials was 30.3% for industry-sponsored and 3.2% for non-industry–sponsored trials. The proportion of international trials conducted in several continents was 60.6% for industry-sponsored and 40.1% for non-industry–sponsored trials. The median number of countries included in international trials was two for industry-sponsored and five for non-industry–sponsored trials.

For industry- and non-industry–sponsored trials, 46.2% and 13.0% of international trials were conducted in at least one Eastern European country, 18.3% and 7.9% in at least one South American country, and 2.4% and 8.9% in at least one African country other than South Africa, respectively (S4 and S5 Figs.).

**Fig 3:**
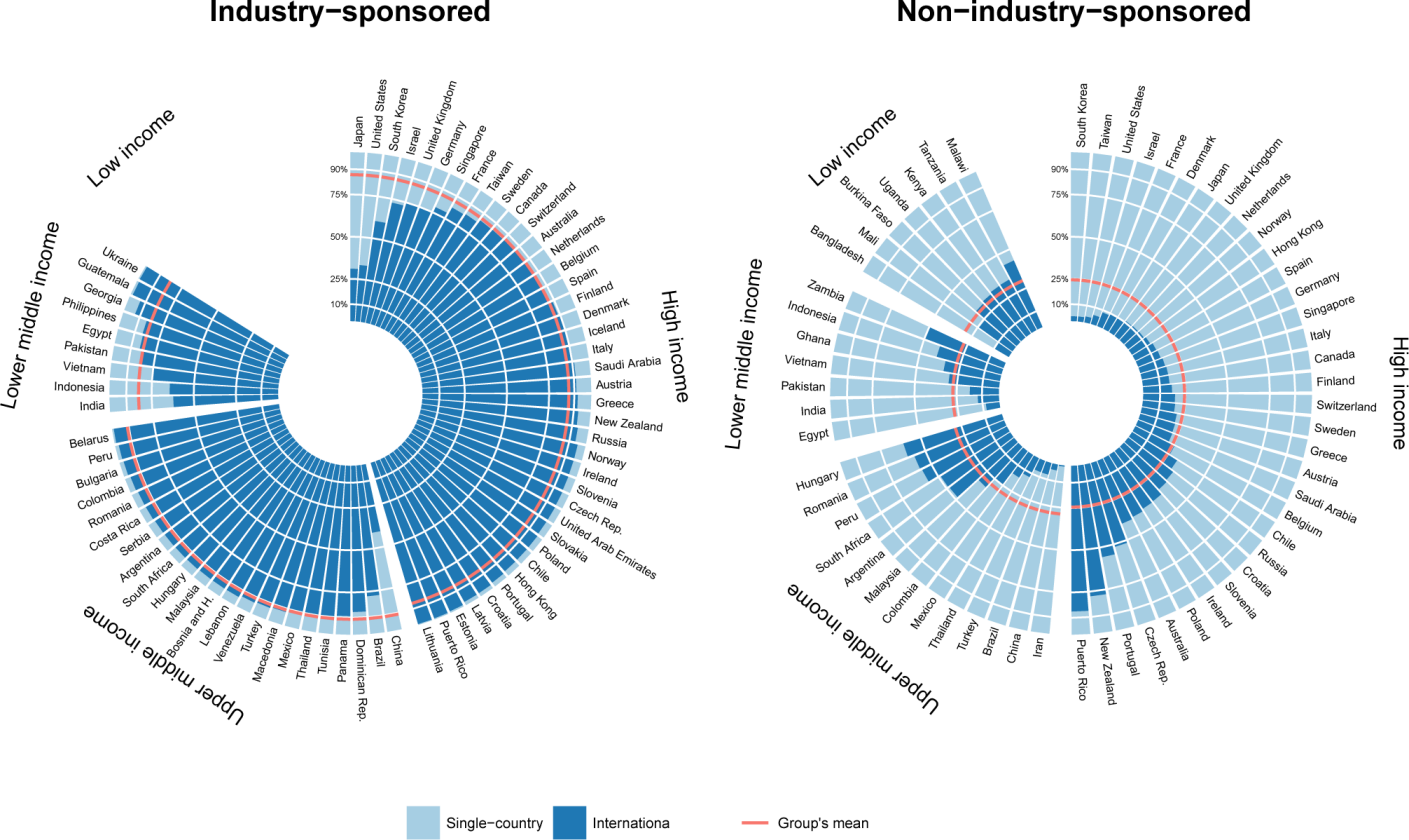
Collaboration ratios of industry- and non-industry–sponsored clinical trials. The radial barplot shows the proportion of international trials (in dark blue) per country for (left) industry- and (right) non-industry–sponsored trials in countries where at least 50 industry- or non-industry–sponsored trials were initiated during the 2006–2013 period. The 72 countries considered for industry- sponsored and the 62 countries considered for non-industry–sponsored trials were grouped by income groups. For each income group and sponsor type, the red line represents the mean proportion of international clinical trials. The exact collaboration ratios per country of industry- and non-industry-sponsored trials per country can be found on S5 and S6 Tables respectively. In all Eastern European and South American countries except Brazil, more than 90% of industry-sponsored research was international, whereas the proportion of international non-industry–sponsored research was lower and more variable, ranging from 25.0% in Colombia to 60.9% in Hungary.

Among industry-sponsored trials, the proportion of international trials in most high-income countries (37 of 40) was more than 70%, and in 21 of these 37 countries, it was greater than 90% (Fig 3). In contrast, among non-industry–sponsored trials, the proportion of international trials in half of the high-income countries was less than 20%. Similar discrepancies were observed for all other income groups.

In high- and upper-middle-income countries, the median number of industry-sponsored international trials per million inhabitants was 10 times that of non-industry–sponsored international trials (61.8 and 6.6 vs. 7.0 and 0.6, respectively). In low-income countries, the 10-fold difference was reversed (0.03 vs. 0.37, respectively).

### Collaboration network analysis

**Fig 4:**
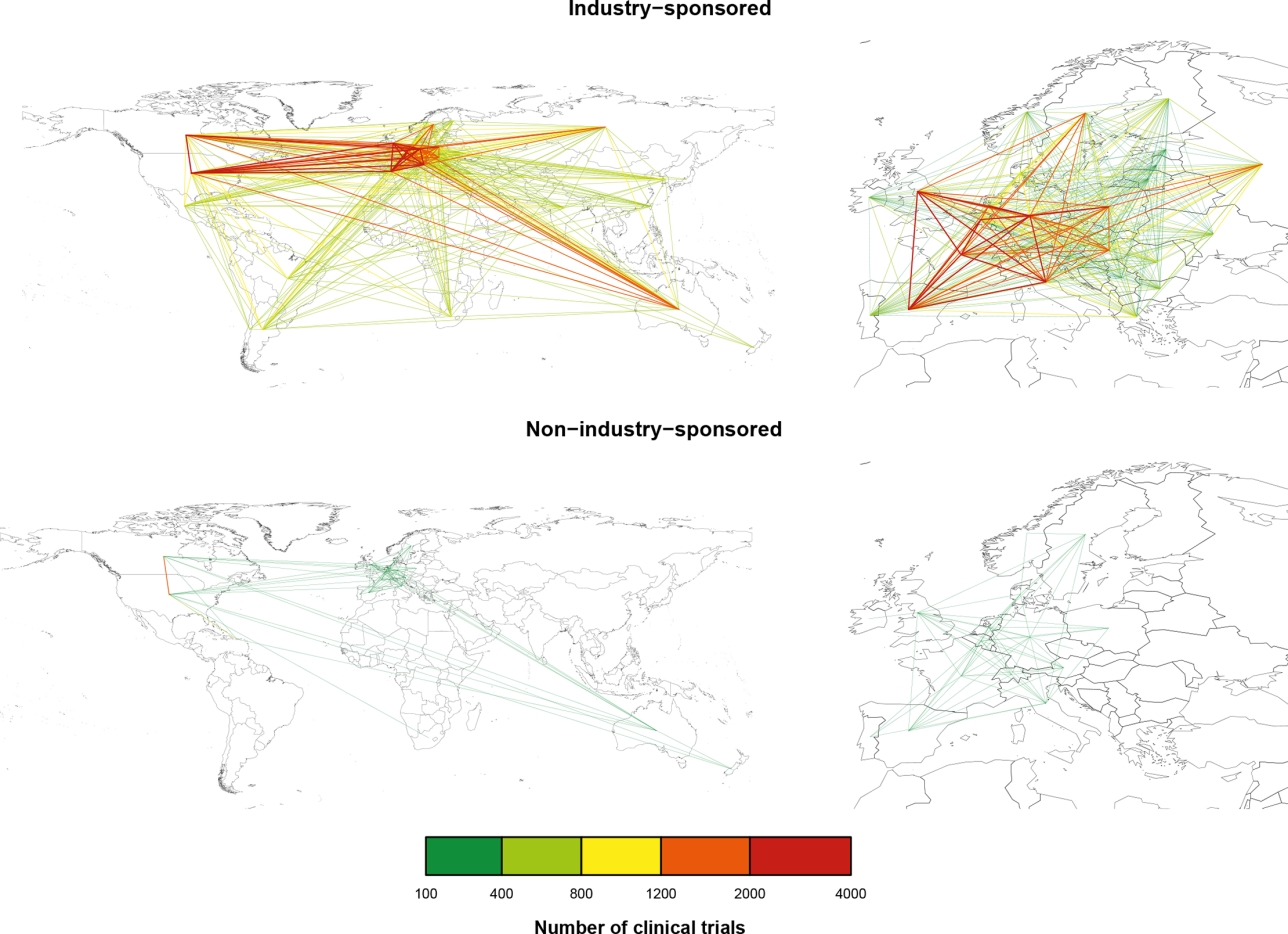
World and European collaboration networks in industry- and non-industry–sponsored clinical trials. Collaboration network of industry- (top) and non-industry–sponsored (bottom) clinical trials for registered trials initiated from 2006 to 2013; the color of a link between two countries corresponds to the number of clinical trials simultaneously conducted in both countries. For clarity, links between 100 and 400 clinical trials are not shown for the world’s industry-sponsored network.

The industry-sponsored network of collaboration included 138 countries and 4,711 country-country collaborations; 613 country-country collaborations accounted for more than 250 trials, with 2,870 trials for the United States–Canada collaboration (Fig 4). The non-industry–sponsored network of collaboration included 154 countries and 3,259 country-country collaborations. The United States–Canada collaboration was the unique collaboration, with more than 250 trials (653 trials). After trimming, the two networks comprised 60 countries (1,770 country-country collaborations) and 65 countries (1,736 country-country collaborations), respectively.

We found 440 (24.9%) and 316 (18.2%) overrepresented collaborations among industry- and non- industry–sponsored country-country collaborations, respectively. The 20 most overrepresented collaborations for industry- and non-industry–sponsored networks were between neighbor countries.

**Fig 5:**
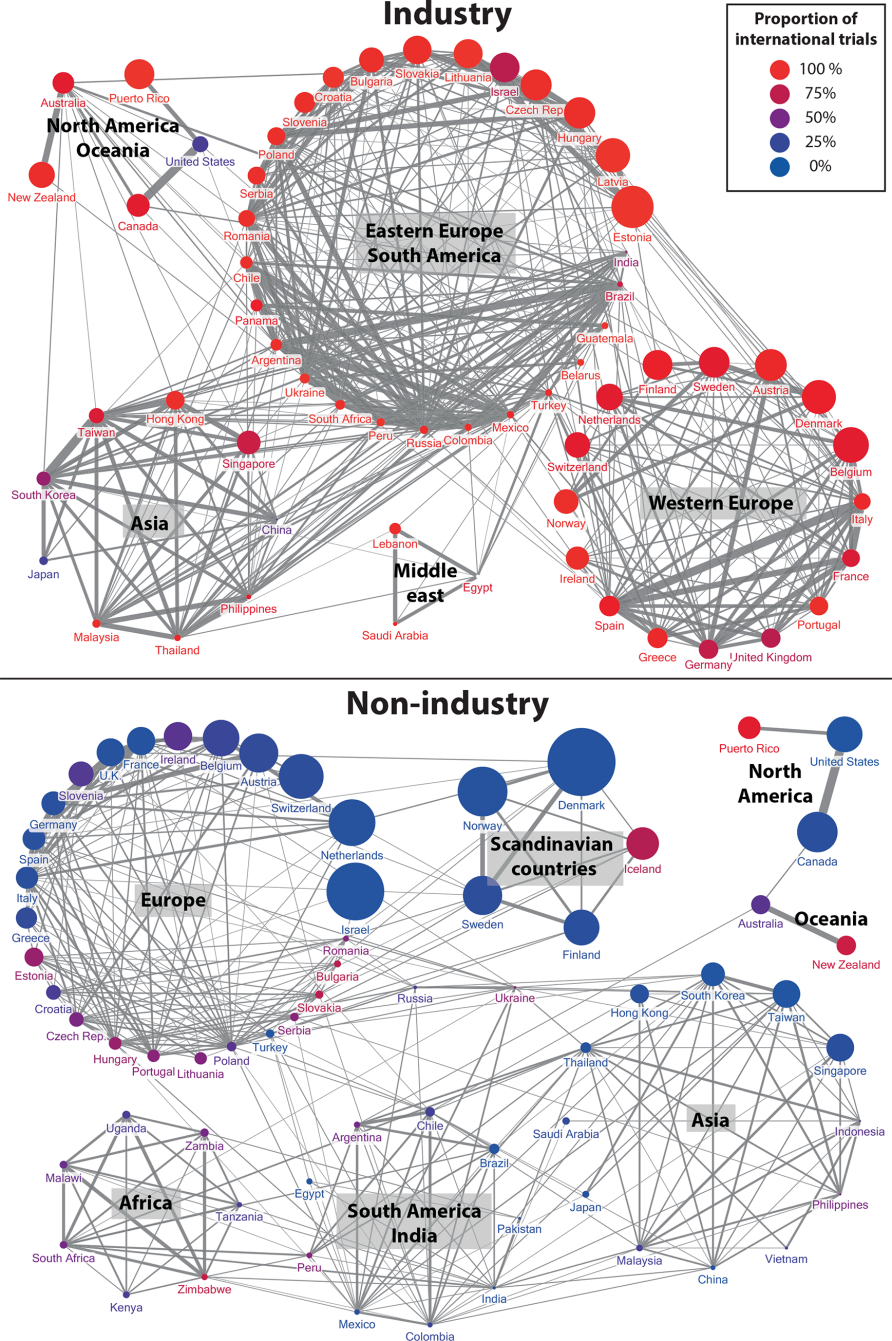
Industry- and non-industry–sponsored co-occurrence networks. Country-country industry- (top) and non-industry–sponsored (bottom) networks for which links between countries are as wide as the estimated overrepresentation of the country-country collaboration. Size of nodes is proportional to the number of (top) industry- or (bottom) non-industry–sponsored clinical trials per million inhabitants. The color of the node represents the collaborative ratio of the country: the color corresponds to a gradient between blue, representing 100% of trials conducted in that country being single-country and red, 100% international trials. Among the 15 industry-sponsored most overrepresented collaborations, three were between Eastern European countries, four between South American countries, two between Asian countries, three between Western European countries (the France–Italy–Spain triangle), and the collaborations United States–Canada and Australia–New Zealand. The 15 most significantly overrepresented non-industry–sponsored collaborations were United States–Canada, United States–Puerto Rico, Australia–New Zealand, Malawi–Zimbabwe, South Korea–Taiwan, three collaborations between Northern European countries and six collaborations between other Western European countries. Among industry-sponsored collaborations, the trimming suppressed all African countries. Among non-industry–sponsored collaborations, African countries did not have overrepresented collaborations with European or North American countries.

Cluster analysis of the co-occurrence networks identified 5 and 8 clusters for industry- and non-industry–sponsored trials, respectively (Fig 5). Most of the clusters corresponded to geographical regions. In the industry-sponsored network, the largest cluster corresponded to South American and Eastern European countries, which were apart from the Western European countries. In the non-industry–sponsored network, Scandinavian countries were clustered apart from the other European countries.

### Evolution over time

**Fig 6:**
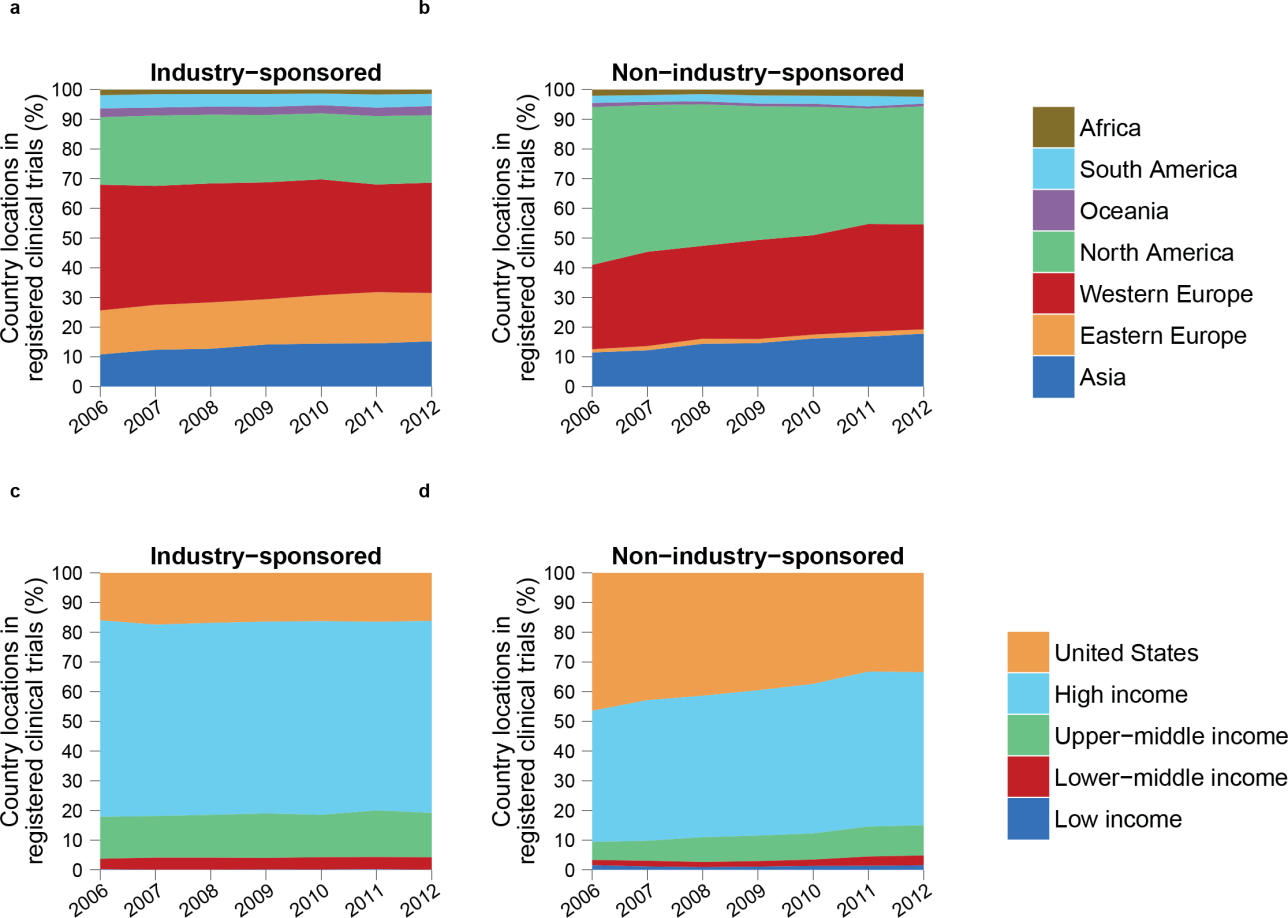
A differentiated global migration of clinical trials on sponsorship. The annual distribution of country trial locations of clinical trials initiated between 2006 and 2012. Countries were grouped by (top) geographical regions and (bottom) income groups, and trials were classified on sponsor type: (left) industry- and (right) non-industry–sponsored. Country-locations of international trials were considered individually: a trial conducted simultaneously in two South American countries would count twice when calculating the share of South America. For income groups, data for the United States and other high-income countries are shown separately. The proportion of trials initiated in Asia increased by a similar amount for both sponsor types (from 10.8% to 15.2% for industry and from 11.5% to 17.8% for non-industry). The proportion of trials initiated in Africa, South America, Oceania, and Eastern Europe remained stable for both sponsor types. The distribution of trials by income groups remained stable for industry-sponsored trials. For non-industry–sponsored trials, the proportion of trials initiated in high-income countries was 90.6% in 2006 and 85.0% in 2012, and the proportion of trials initiated in upper-middle-income countries was 6.1% in 2006 and 10.2% in 2012. The proportion of lower-middle- and low-income groups remained stable for non- industry–sponsored trials. The exact share of country trial locations of industry- and non-industry-sponsored trials per geographic region can be found on S7 and S8 Tables respectively. The exact share of country trial locations of industry- and non-industry-sponsored trials per income group can be found on S9 and S10 Tables respectively.

Overall, the number of trials increased in all regions and income groups between 2006 and 2012. The distribution of trials over geographical regions and over income groups evolved differently when comparing sponsors (Fig 6). In particular, for industry-sponsored trials, the proportion of trials initiated in Western Europe was 42.3% in 2006 and 37.1% in 2012 and for non-industry–sponsored trials was 28.4% in 2006 and 35.3% in 2012. Conversely, the proportion of trials initiated in North America remained stable for industry-sponsored trials (23.1% on average) but was 53.1% in 2006 and 39.7% in 2012 for non-industry–sponsored trials.

In total, the proportion of industry-sponsored trials was 32.9% in 2006 and 28.8% in 2012. This decrease was not equivalent in all geographical regions and income groups (S2 and S3 Tables). The proportion of industry-sponsored trials decreased by approximately 20% between 2006 and 2012 in Africa but remained stable in North America.

The number of international trials increased over the study period for both sponsor types. Meanwhile, the share of international trials among all trials decreased for both sponsor types (from 34.2% to 29.1% and from 3.6% to 2.9% for industry- and non-industry–sponsored trials, respectively). For industry-sponsored trials, the proportion of international trials conducted in several continents was 54.8% in 2006 and 67.3% in 2012 but for non-industry–sponsored trials was 42.4% in 2006 and 37.2% in 2012.

## Discussion

In this bird's eye analysis of all registered clinical trials that were included in the WHO ICTRP, we found that clinical trials were unequally distributed in the world. Sponsorship has a major influence in this unequal mapping. International collaboration in clinical trials was mainly used by industry-sponsors, while non-industry–sponsored trials were mainly conducted in a single country.

Clinical trials were particularly prevalent in high-income countries and Eastern Europe and lacking in low-income countries. We documented substantial gaps in the global distribution of clinical trials between industry- and non-industry–sponsored research. Most of the clinical trials conducted in Eastern Europe were industry-sponsored but in Africa were non-industry–sponsored. International collaboration was sparse for academic sponsors, with 97% of academic-sponsored trials conducted in a single country. International collaboration was mainly used by industry sponsors in well-defined networks such as Eastern Europe and South America. In these regions, few single-country trials were conducted, so these countries may not conduct their own clinical trials. International trials were mainly conducted between neighboring countries, but the groups of countries that collaborated differed for both sponsor types.[29] The locations of industry-sponsored trials remained stable since 2006, whereas non-industry–sponsored trial locations showed a shift from North America to Western European and Asian countries. More international trials were conducted over time, but the share of international trials among all clinical trials decreased for both sponsor types. In addition, industry-sponsored international trials became more inter-continental over time.

Clinical research is needed globally to validate treatment efficacy in the broadest population, to find local answers where universal questions may not be valid, and to improve health systems in emerging economies, but the location of clinical trials depends on sponsor’s strategies and constraints.[5] Recently, Drain et al showed the unequal distribution and the global migration of clinical trials but did not study the impact of sponsorship.[12] Previous studies showed the unequal mapping of industry-sponsored clinical trials.[11,30] Our results are in line with these previous results and add to the substantial influence of sponsorship in the unequal global distribution of clinical trials and its evolution. The differential patterns of collaboration between the two sponsor types may underlie differentiated strategies and constraints in conducting international trials. In particular, academic sponsors may not have the operational and financial capacities to conduct trials worldwide, whereas industry sponsors may have more economical reasons to conduct international trials in specific regions such as Eastern Europe and South America. Initiatives have attempted to enhance academic collaboration networks. For instance, the European Clinical Research Infrastructures Network aims at promoting collaborative clinical research in Europe.[31] As well, the new European Union regulation on clinical research adopted last year modified the procedures for the authorization of clinical trials in order to stimulate international research.[32] Other initiatives attempt to favor trials between European and African countries.[33] Nevertheless, the number of international clinical trials conducted by non-industry sponsors still remains extremely low as compared to those conducted by the pharmaceutical industry. The future replication of these analyses would allow monitoring research agendas and assess the impact of initiatives or regulations aiming to stimulate international research.

The principal strengths of our study are the global overview of the system of clinical trials and the complex systems approach to analyse the system of international trials. However, the limitations are the self-reported nature of clinical trial registries data and the heterogeneity of what is considered a trial.[18,34] In particular, registries cannot verify the veracity of input trial information. There could be discrepancies between declared trial sites and sites that actually enrolled patients. However, the absence of verification concerns both industry-sponsored and non-industry-sponsored trials. In addition, our analysis is restricted to registered clinical trials. Not all registered clinical trials can be considered as means to increase clinical knowledge. Some clinical trials are conducted only for registration purposes, and several phase IV trials may be conducted for marketing purposes.[35] In addition, the vast majority of observational studies are not prospectively registered and so are consequently not covered by our analyses.[36–38] Moreover, the compliance with clinical trial registration may vary across countries and may be lower in low- and middle-income countries. Clinical trials sponsored by local sources (predominantly non-industry sponsors) and conducted in a single country in these regions may be less likely to be registered. In such a case, our findings regarding the share of single-country non-industry-sponsored clinical trials could be considered conservative. Other sources would allow us to recover data about unregistered trials, as publications registered in bibliographical databases, data from regional R&D hubs, or funders' databases. However, these additional resources are not readily usable nor accessible. In addition, unregistered trials are unlikely to change the gap we found between the proportion of international clinical trials between industry- and non-industry-sponsored trials.

In this work, we chose to categorise sponsorship according to the primary sponsor, the primary organization that has the responsibility of the conduct of the clinical trial. We consider that the primary sponsor would be the most likely to enable or promote the conduct of international trials. One limitation is that we could not analyse the trial funding because such data are not reported in the WHO ICTRP. Sponsorship may not be a perfect proxy for funding research in that companies may influence steps of clinical research other than by sponsorship.[39] However, this situation would mainly concern non-industry-sponsored trials because industry-sponsored trials have high chance of being funded by the industry, but non-industry-sponsored trials may also be (partially) funded by industry.

Some of our choices of thresholds may have affected our results, such as the inclusion of countries for analyses. Nevertheless, these choices were unlikely to change our findings because of the magnitude of the discrepancies we found in analyses by sponsor type. Another limitation is the restriction to the 2006–2012 period for the time evolution analysis, but we considered that we did not have a reliable scope of global mapping outside that period. Another limitation that is not considered in our mapping is the country- or region-specific health needs. For instance, different areas of research may be more likely to motivate international collaboration, in particular in non-industry-sponsored settings.[40–43]. The next step will be to assess whether registered clinical trials correspond to health needs assessed locally.[44] Finally, we did not consider the number of patients included in each trial, which could result in more accurate measures of the amount of research in the population. The target sample size can be extracted from the trial registries but may not exactly correspond to the real sample size, and we have no information on the country-level sample size for international trials. Industry sponsors may have more capacity to conduct larger trials than academic researchers, which may increase the existing gaps between the mappings of both sponsor types.

The collaboration network analysis sheds light on the groups of countries that were more likely to be included together in international clinical trials. However, countries nearby in the collaboration network do not necessarily have scientific or logistic expertise to collaborate in international clinical trials. The weight and nature of the collaboration between countries participating in the same international trials may depend on the will of the primary sponsor to simply outsource the recruitment of patients or the entire conduct of the clinical trial. From the perspective of external validity, the physical location of trial sites does clearly mean that the trial is international. If a trial is performed in multiple geographical regions, one can assess whether the treatment effect is similar or heterogeneous across these settings.

This work is in-line with a series of works aiming to create a global observatory of health research.[8,9] The WHO ICTRP is the single source allowing a bird's-eye view of the mapping of clinical trials.[18,45] Acknowledging all the limitations of clinical trial data and the WHO ICTRP, the substantial gaps we show between the mappings of industry- and non-industry-sponsored trials and collaboration networks are unlikely to be changed. In conclusion, clinical trials are unequally distributed in the world. Substantial gaps exist between the mappings of industry- and non-industry–sponsored trials. International collaboration is lacking in academic-sponsored trials but is a predominant feature of industry-sponsored trials in well-defined networks of countries.

## Supporting Information

S1 Appendix Data extraction and Sponsor classification. (DOCX)

S1 Dataset Minimal dataset. (CSV)

S1 Fig Flowchart. (PDF)

S2 Fig Distribution of clinical trials and population per geographical regions. For each geographic region, the size of the green (blue, respectively) area is proportional to the number of industry- (non-industry–, respectively) sponsored trials initiated during the 2006–2013 period, and the size of the red area is proportional to the population as of 2012. Equal sized trial and population squares correspond to an overall density of 10 trials per million inhabitants. The proportion of industry-sponsored clinical trials was 57.0%, 37.5%, 51.0%, 92.5%, 65.4%, 77.2% and 45.8% in Western Europe, North America, Asia, Eastern Europe, South America, Oceania and Africa, respectively.
(PDF)

S3 Fig Mapping of single-country for industry- and non-industry–sponsored clinical trials. The number of single-country clinical trials per million inhabitants for industry-sponsored (top) and non-industry–sponsored (bottom) research for registered trials initiated between 2006 and 2013.
(PDF)

S4 Fig Mapping of international trials for industry- and non-industry–sponsored clinical trials. The number of international clinical trials per million inhabitants for industry-sponsored (top) and non-industry–sponsored (bottom) research for registered trials initiated between 2006 and 2013.
(PDF)

S5 Fig Mapping of single-country and international clinical trials for industry- and non-industry–sponsored clinical trials in Europe. The number of single-country (top) and international (bottom) clinical trials per million inhabitants for industry-sponsored (left) and non-industry–sponsored (rigth) research for registered trials initi- ated between 2006 and 2013 in Europe.
(PDF)

S1 Table Summary of the number of registered trials initiated in 2006–2013 per million inhabitants per geographical region. (PDF)

S2 Table Proportion of industry-sponsored trials per year for each geographical region. (PDF)

S3 Table Proportion of industry-sponsored trials per year for each income group. (PDF)

S4 Table Proportion of industry-sponsored trials for each country. (PDF)

S5 Table Proportion of international clinical trials among industry-sponsored trials for each country. (PDF)

S6 Table Proportion of international clinical trials among non-industry-sponsored trials for each country. (PDF)

S7 Table Distribution of country trial location of industry-sponsored trial over geographical regions per year. (PDF)

S8 Table Distribution of country trial location of non-industry-sponsored trial over geographical regions per year. (PDF)

S9 Table Distribution of country trial location of industry-sponsored trial over income groups per year. (PDF)

S10 Table Distribution of country trial location of non-industry-sponsored trial over income groups per year. (PDF)

## References

[1] Pang T, Terry RF, The PLoS Medicine Editors. Who/PLoS Collection “No Health Without Research”: A Call for Papers. PLoS Med. 2011;8: 1–2. 10.1371/journal.pmed.1001008

[2] Dye C, Boerma T, Evans D, Harries A, Lienhardt C, McManus J, et al Research for universal health coverage. Sci Transl Med. 2013;5: 1–146. 10.1126/scitranslmed.3006971 23966297

[3] Søreide K, Alderson D, Bergenfelz A, Beynon J, Connor S, Deckelbaum DL, et al Strategies to improve clinical research in surgery through international collaboration. Lancet. 2013;382: 1140–1151. 10.1016/S0140-6736(13)61455-5 24075054

[4] OECD. Facilitating International Cooperation in Non-Commercial Clinical Trials OECD Glob Sci Forum 2011;

[5] Lang T, Siribaddana S. Clinical trials have gone global: Is this a good thing? PLoS Med. 2012;9: 6 10.1371/journal.pmed.1001228 PMC3373653 22719228

[6] Trimble EL, Abrams JS, Meyer RM, Calvo F, Cazap E, Deye J, et al Improving Cancer Outcomes Through International Collaboration in Academic Cancer Treatment Trials. J Clin Oncol. 2009;27: 5109–5114. 10.1200/JCO.2009.22.5771 19720905 PMC2799058

[7] Chalmers I, Bracken MB, Djulbegovic B, Garattini S, Grant J, Gülmezoglu AM, et al How to increase value and reduce waste when research priorities are set. Lancet. 2014;383: 156–165. 10.1016/S0140-6736(13)62229-1 24411644

[8] Terry RF, Salm JF, Nannei C, Dye C. Creating a global observatory for health R&D. Science. 2014;345: 1302–1304. 10.1126/science.1258737 25214621

[9] Røttingen JA, Regmi S, Eide M, Young AJ, Viergever RF, Ardal C, et al Mapping of available health research and development data: what’s there, what's missing, and what role is there for a global observatory? Lancet. 2013;382: 1286–1307. 10.1016/S0140-6736(13)61046-6 23697824

[10] Glickman SW., McHutchison JG., Peterson ED. et al Ethical and Scientific Implications of the Globalization of Clinical Research. N Engl J Med. 2009;360: 816–23. 10.1056/NEJMsb0803929 19228627

[11] Thiers F a., Sinskey AJ, Berndt ER. Trends in the globalization of clinical trials. Nat Rev Drug Discov. 2008;7: 13–14. 10.1038/nrd2441

[12] Drain PK, Robine M, Holmes KK, Bassett I V, Clinical I, Registry T, et al Trail watch: global migration of clinical trials. Nat Rev Drug Discov. 2014;13: 166–7. 10.1038/nrd4260 24577390 PMC4006355

[13] Evans J a, Shim J-M, Ioannidis JP a. Attention to local health burden and the global disparity of health research. PLoS One. 2014;9: e90147 10.1371/journal.pone.0090147 24691431 PMC3972174

[14] Viergever RF, Terry RF, Karam G. Use of data from registered clinical trials to identify gaps in health research and development. Bull World Heal Organ. 2013; 416–425C. 10.2471/BLT.12.114454 PMC3777147 24052678

[15] Patsopoulos N a, Ioannidis JP a, Analatos A a. Origin and funding of the most frequently cited papers in medicine: database analysis. BMJ. 2006;332: 1061–1064. 10.1136/bmj.38768.420139.80 16547014 PMC1458565

[16] Justin Chakma, B. Sc., Gordon H. Sun, M.D., Jeffrey D. Steinberg, Ph.D., Stephen M. Sammut, M.A. MBA, and Reshma Jagsi, M.D. DP. Asia’s Ascent—Global Trends in Biomedical R&D Expenditures. N Engl J Med. 2013; 3–6. 10.1056/NEJMp1313927 24382062

[17] WHO. International Clinical Trials Registry Platform [Internet]. Available: http://apps.who.int/trialsearch/

[18] Dickersin K, Rennie D. The evolution of trial registries and their use to assess the clinical trial enterprise. JAMA. 2012;307: 1861–4. 10.1001/jama.2012.4230 22550202

[19] Wick M. GeoNames. Symposium on Space-Time Integration in Geography and GIScience. 2011.

[20] Lazzeri V. Eurovoc. Terminol. 1983; 31–35.

[21] Connor EF, Simberloff D. The assembly of species communities—chance or competition. Ecology. 1979;60: 1132–1140. 10.2307/1936961

[22] Gotelli NJ. Null model analysis of species co-occurrence patterns. Ecology. 2000;81: 2606–2621. 10.1890/0012-9658(2000)081[2606:NMAOSC]2.0.CO;2

[23] Oksanen J, Blanchet FG, Kindt R, Legendre P, Minchin PR, O’Hara RB, et al Package “vegan.” R Packag ver 20–8. 2013; 254.

[24] Rosvall M, Bergstrom C. Maps of random walks on complex networks reveal community structure. Proc Natl Acad Sci. 2007;105: 1118–23. 10.1073/pnas.0706851105 18216267 PMC2234100

[25] Viergever RF, Karam G, Reis A, Ghersi D. The Quality of Registration of Clinical Trials: Still a Problem. 2014;9: 12–20. 10.1371/journal.pone.0084727 PMC3888400 24427293

[26] R Development Core Team. R: A Language and Environment for Statistical Computing [Internet]. R Foundation for Statistical Computing Vienna Austria 2013 pp. {ISBN} 3–900051–07–0. Available: http://www.r-project.org/

[27] Edler D, Rosvall M. The MapEquation software package, available online at http://www.mapequation.org. 2013.

[28] Hansen DL, Shneiderman B, Smith M a. Analyzing Social Media Networks with NodeXL. Anal Soc Media Networks with NodeXL. 2011; 11–29 10.1016/B978-0-12-382229-1.00002-3

[29] Pan RK, Kaski K, Fortunato S. World citation and collaboration networks: uncovering the role of geography in science. Sci Rep. 2012;2: 1–7. 10.1038/srep00902 PMC3509350 23198092

[30] Karlberg JPE. Correspondence—Globalization of sponsored clinical trials. Nat Rev Drug Discov. 2007;2007: 1–3.

[31] Demotes-Mainars J, Ohmann C. European Clinical Research Infrastructures Network: promoting harmonisation and quality in European clinical research. Lancet. 2005;365: 107–108. 15639280 10.1016/S0140-6736(05)17720-4

[32] European Parliament and Council of the European Union. Regulation (EU) No 536/2014 of the European Parliament and of the Council of 16 April 2014 on clinical trials on medicinal products for human use, and repealing Directive 2001/20/EC. Off J Eur Union. 2014;L: 1–76. Available: http://eur-lex.europa.eu/legal-content/EN/TXT/PDF/?uri=OJ:JOL_2014_158_R_0001&from=EN. Accessed 14 July 2014.

[33] Matee MI, Manyando C, Ndumbe PM, Corrah T, Jaoko WG, Kitua AY, et al European and Developing Countries Clinical Trials Partnership (EDCTP): the path towards a true partnership. BMC Public Health. 2009;9: 249 10.1186/1471-2458-9-249 19619283 PMC2719636

[34] Viergever RF, Li K. Trends in global clinical trial registration: an analysis of numbers of registered clinical trials in different parts of the world from 2004 to 2013. 2015; 1–16. 10.1136/bmjopen-2015-008932 PMC4593134 26408831

[35] Sox HC, Rennie D. Seeding trials: just say “no”. Annals of internal medicine. 2008. pp. 279–280. VL—149 10.7326/0003-4819-149-4-200808190-00012 18711161

[36] Editors TPM. Observational Studies: Getting Clear about Transparency. PLoS Med. 2014;11: e1001711 10.1371/journal.pmed.1001711 25158064 PMC4144975

[37] Dal-Re R, Ioannidis JP, Bracken MB, Buffler PA, Chan AW, Franco EL, et al Making prospective registration of observational research a reality. Sci Transl Med. 2014;6: 224cm1 10.1126/scitranslmed.3007513 24553383

[38] Boccia S, Rothman KJ, Panic N, Flacco ME, Rosso A, Pastorino R, et al Registration practices for observational studies on clinicaltrials.gov indicated low adherence. J Clin Epidemiol. Elsevier Ltd; 2015; 10.1016/j.jclinepi.2015.09.009 26386325

[39] Stamatakis E, Weiler R, Ioannidis JPA. Undue industry influences that distort healthcare research, strategy, expenditure and practice: a review. 2010; 1–7. 10.1111/eci.12074 23521369

[40] Sista ND, Karim QA, Hinson K, Donnell D, Eshleman SH, Vermund SH. Experience in international clinical research: The HIV Prevention Trials Network. Clin Investig (Lond). 2011;1: 1609–1618. 10.4155/cli.11.156 PMC3281583 22348195

[41] International Maternal Pediatric Adolescent AIDS Clinical Trials Network (IMPAACT) [Internet]. Available: impaactnetwork.org/index.htm

[42] HIV Vaccine Trials Network (HVTN) [Internet]. Available: http://www.hvtn.org/en.html

[43] AIDS Clinical Trials Group (ACTG) [Internet]. Available: https://actgnetwork.org/

[44] Emdin C a, Odutayo A, Hsiao AJ, Shakir M, Hopewell S, Rahimi K, et al Association between randomised trial evidence and global burden of disease: cross sectional study (Epidemiological Study of Randomized Trials—ESORT). BMJ. 2015;350 10.1136/bmj.h117 PMC4309646 25630558

[45] Weber WEJ, Merino JG, Loder E. Trial registration 10 years on. BMJ. 2015;3572: h3572 10.1136/bmj.h3572 26149708

