# Supplementary material for: Differential Globalization of Industry- and Non-Industry–Sponsored Clinical Trials
Source: PLoS One. 2015 Dec 14;10(12):e0145122. doi: 10.1371/journal.pone.0145122 (PMC4681996; doi:10.1371/journal.pone.0145122)
Supplement: S1 Appendix — (DOCX) [file pone.0145122.s001.docx]

Appendix

# Trial data extraction and management

# All trial registries were extracted in XML format after empty searches from ClinicalTrials.gov and from WHO International Clinical Trials Registry Platform on February 2, 2014. Data was managed using R software.

# For each trial we extracted the following fields present in the XML documents:

# Main ID

# Secondary ID

# Primary sponsor

# Sponsor type (only in ClinicalTrials.gov registries)

# Country location(s)

# Start date

# Duplicates were identified using the Main ID and Secondary ID fields.

# Sponsor classification

# Trial sponsors that did not appear in the sponsor list of ClinicalTrials.gov were classified as industry or non-industry if one of the following keywords was included in the sponsor name (case sensitive search).

# Keywords for industry sponsors: "Inc", "INC", "LTD", "Limited", "LIMITED", "Ltd", "Co.", "Corporation", "Company", "LLC", "S.A", "A/S", "a.s", "S A", "S. A", "S.p.A", "S.L", "S. L", "SAS", "GmbH",

# "GMBH", "Pvt", "Private", "Pharma", "pharma", "PHARMA", "ROCHE", "Praxis", "Laborato", "LABORATO", "Plc", "plc", "FARMEX".

# Keywords for non-industry sponsors: "institut", "Institut", "INSTITUT", "Univ", "univ", "UNIV", "UMC", "UH", "Uniklinik", "College", "COLLEGE", "college", "World Health Organization", "World health Organization", "NHS", "Public", "UCL", "Hospital", "HOSPITAL", "Hospices", "hospices", "Hôpital", "hospital", "Hopital", "World", "WORLD", "national", "National", "Social", "Zon", "Minist", "Foundation", "Fundación", "FONDAZIONE", "Fondation", "Medical Research Council", "MRC", "School", "Klinik", "ISTITUT", "Istitut", "UZ", "Medical Center", "Medisch Centrum", "Council", "council", "COUNCIL", "Grupo Español", "GRUPO ESPAÑOL", "Grupo de ", "GRUPO DE", "GRUPPO ITALIANO",

# "Pública", "Fédération", "Facul", "Research Center", "Research Centre", "research center", "research centre", "Zentrum", "Wellcome Trust", "medical center", "Medical center".

# When the algorithm classified a trial as both industry- and non-industry–sponsored (209/15273 trials), the sponsor was screened and manually classified.
