# Supplementary figures and images for: Differential Globalization of Industry- and Non-Industry–Sponsored Clinical Trials
Source: PLoS One. 2015 Dec 14;10(12):e0145122. doi: 10.1371/journal.pone.0145122 (PMC4681996; doi:10.1371/journal.pone.0145122)

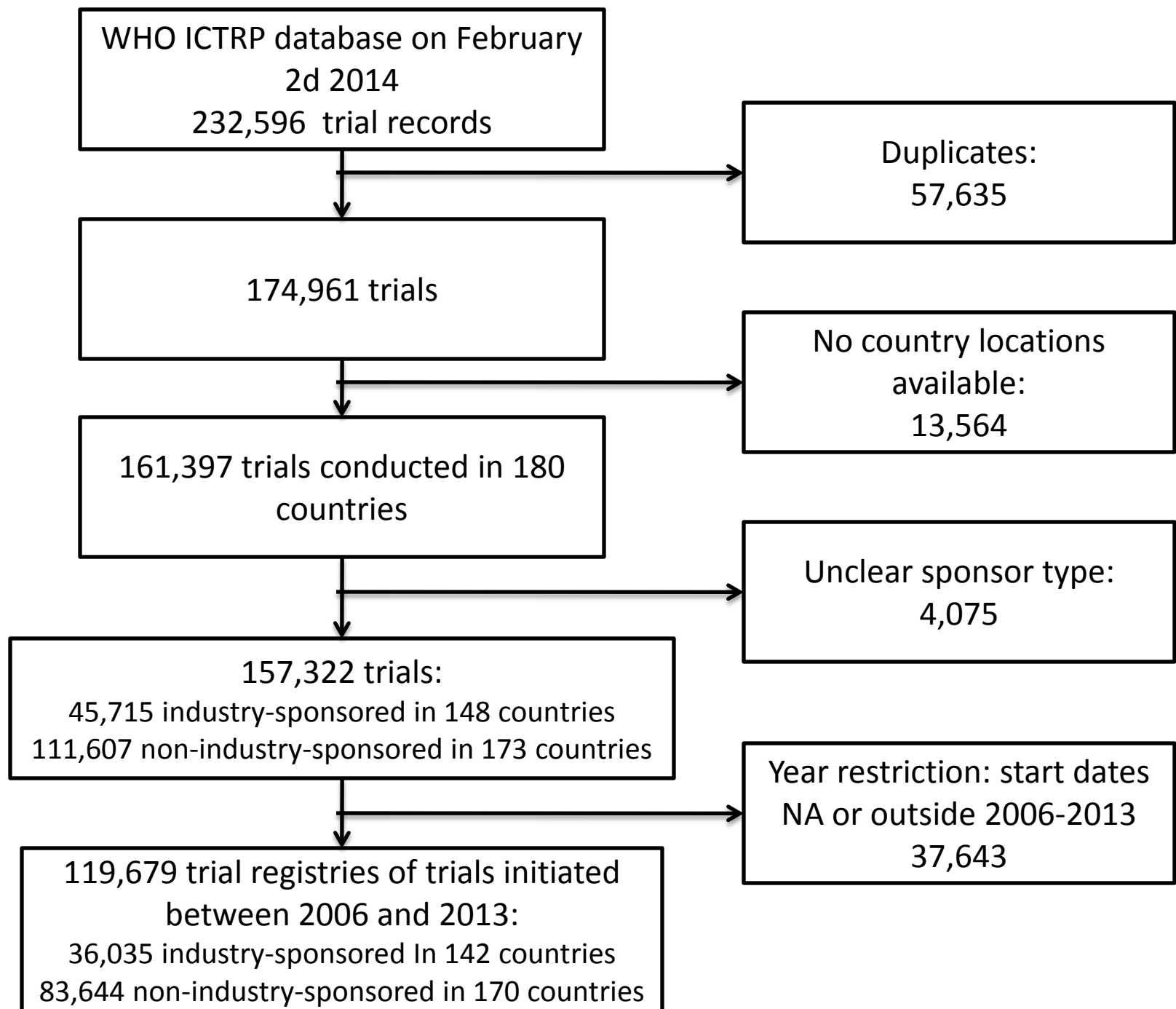

Supplement: S1 Fig — (PDF) [file pone.0145122.s003.pdf]

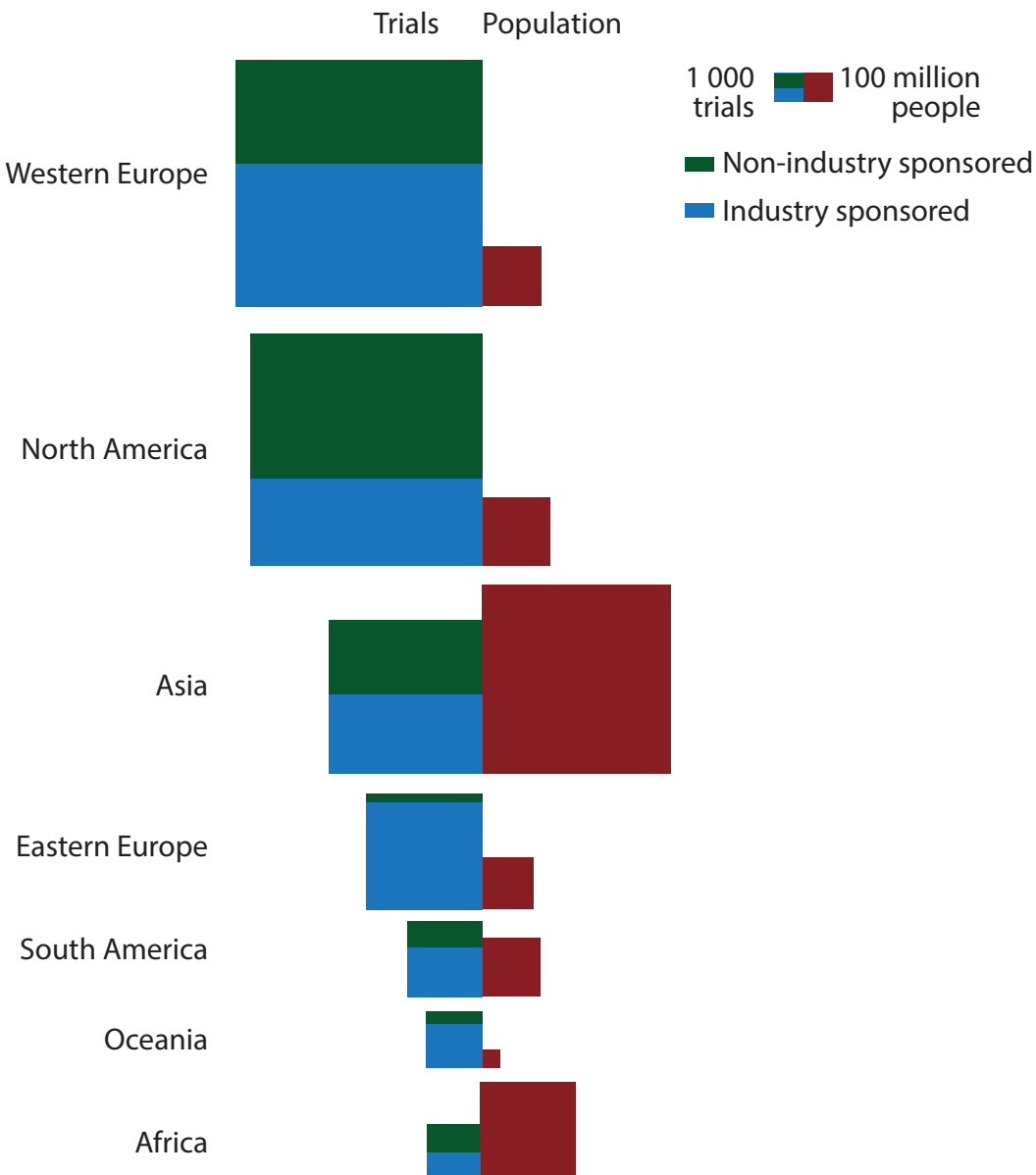

Supplement: S2 Fig — For each geographic region, the size of the green (blue, respectively) area is proportional to the number of industry- (non-industry–, respectively) sponsored trials initiated during the 2006–2013 period, and the size of the red area is proportional to the population as of 2012. Equal sized trial and population squares correspond to an overall density of 10 trials per million inhabitants. The proportion of industry-sponsored clinical trials was 57.0%, 37.5%, 51.0%, 92.5%, 65.4%, 77.2% and 45.8% in Western Europe, North America, Asia, Eastern Europe, South America, Oceania and Africa, respectively. (PDF) [file pone.0145122.s004.pdf]

## Industry

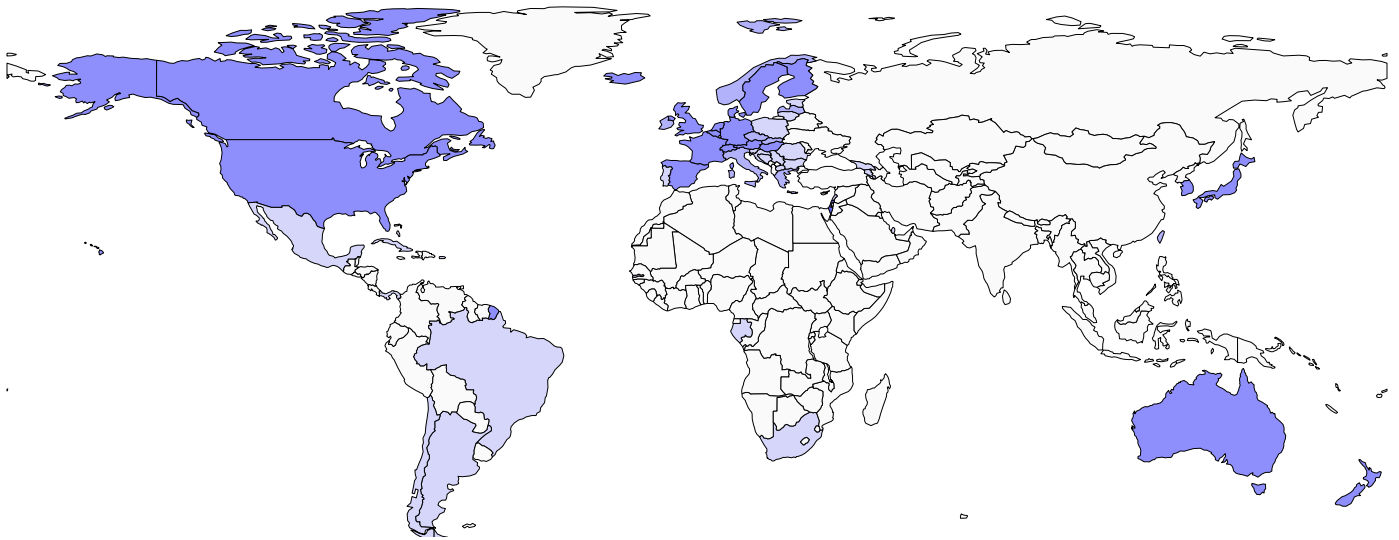

## Non-industry

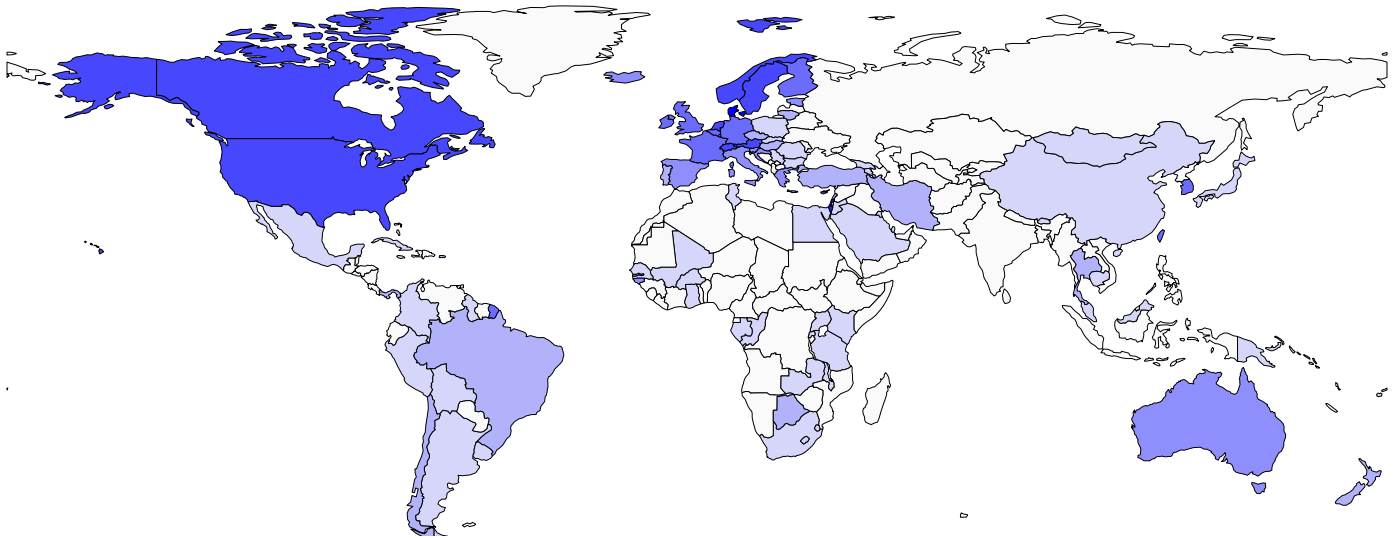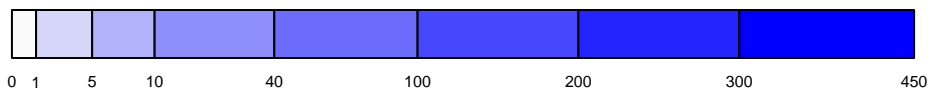

Number of single-country clinical trials per 1 million inhabitants

Supplement: S3 Fig — The number of single-country clinical trials per million inhabitants for industry-sponsored (top) and non-industry–sponsored (bottom) research for registered trials initiated between 2006 and 2013. (PDF) [file pone.0145122.s005.pdf]

## Industry

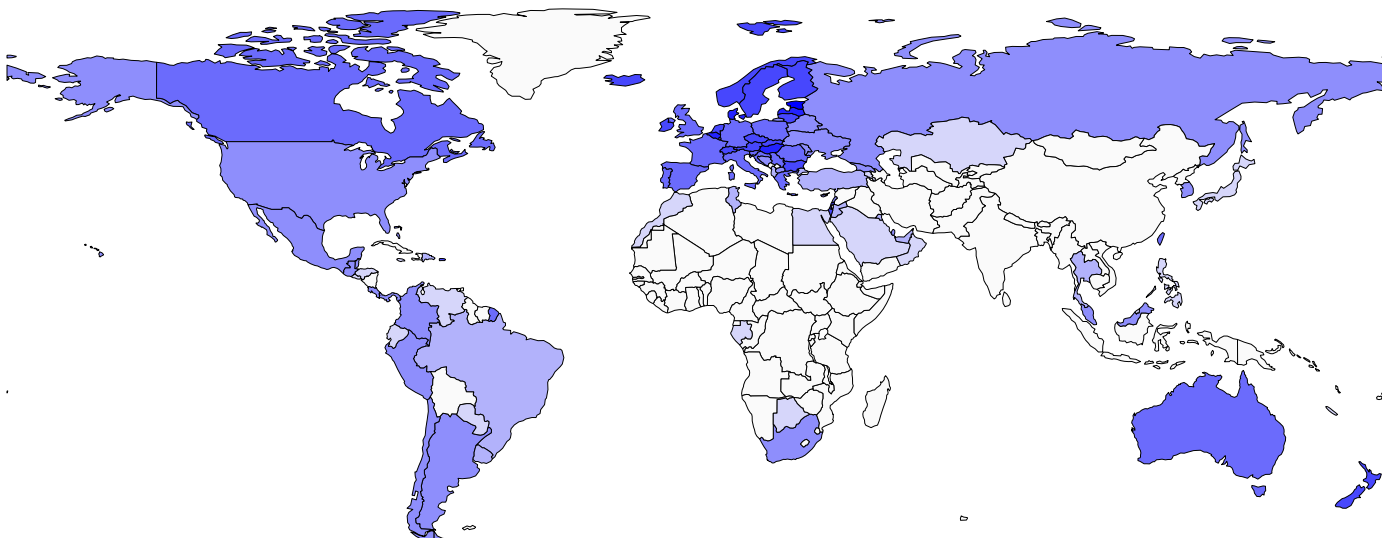

## Non-industry

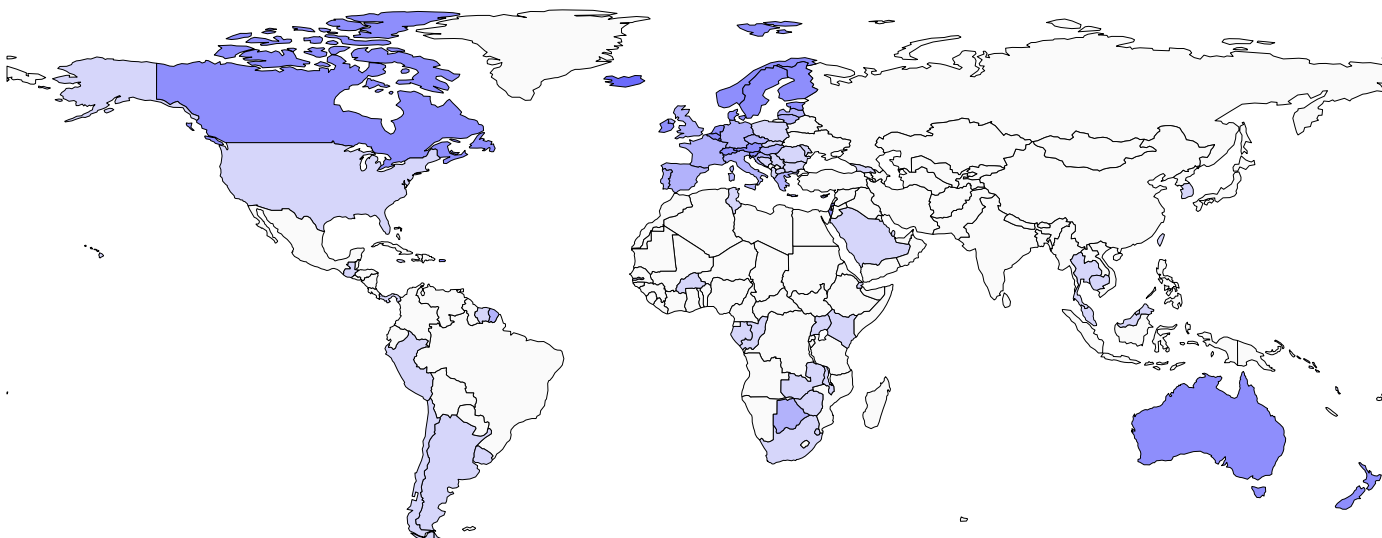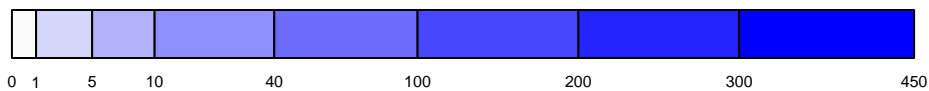

Number of international clinical trials per 1 million inhabitants

Supplement: S4 Fig — The number of international clinical trials per million inhabitants for industry-sponsored (top) and non-industry–sponsored (bottom) research for registered trials initiated between 2006 and 2013. (PDF) [file pone.0145122.s006.pdf]
