# Supplementary material for: Differential Globalization of Industry- and Non-Industry–Sponsored Clinical Trials
Source: PLoS One. 2015 Dec 14;10(12):e0145122. doi: 10.1371/journal.pone.0145122 (PMC4681996; doi:10.1371/journal.pone.0145122)
Supplement: S5 Fig — The number of single-country (top) and international (bottom) clinical trials per million inhabitants for industry-sponsored (left) and non-industry–sponsored (rigth) research for registered trials initi- ated between 2006 and 2013 in Europe. (PDF) [file pone.0145122.s007.pdf]

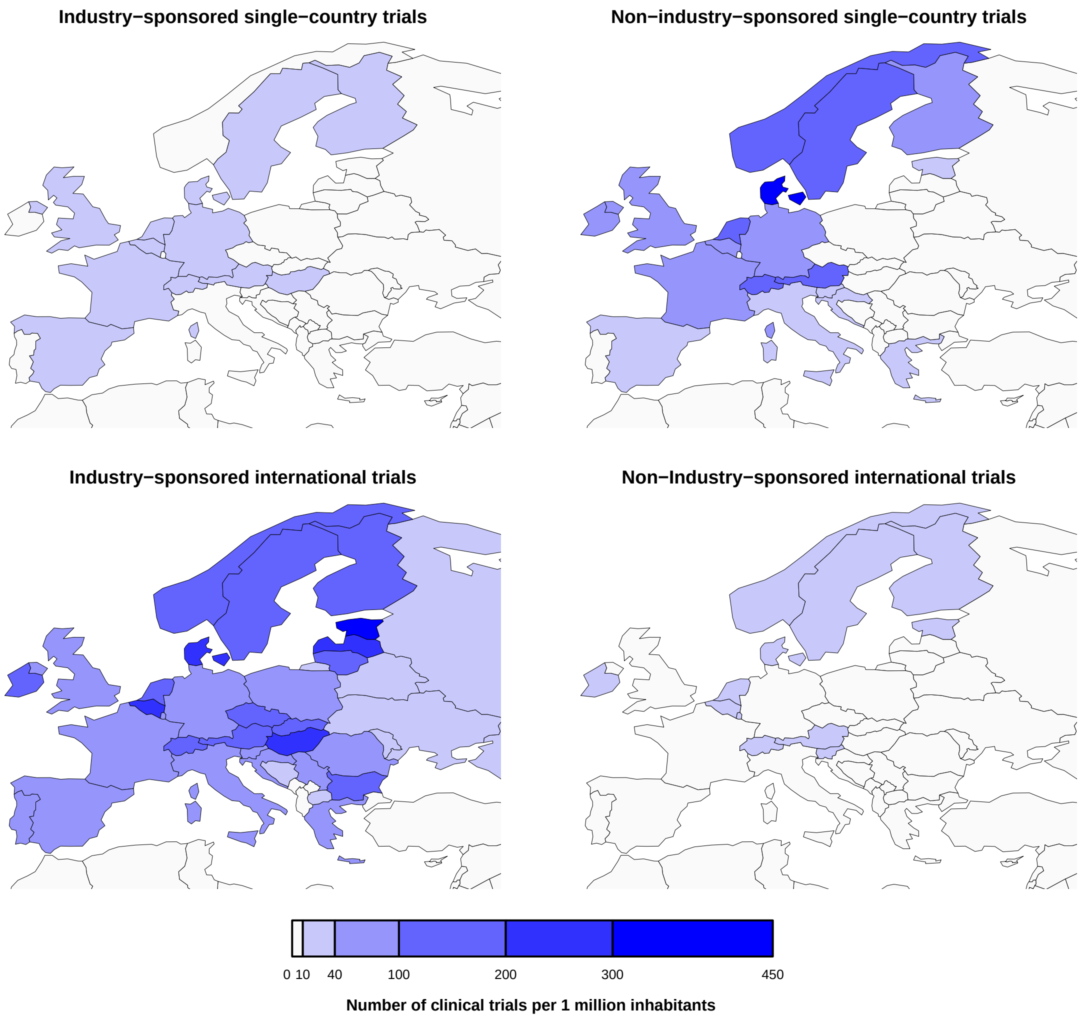

Industry−sponsored single−country trials
Non−industry−sponsored single−country trials
Industry−sponsored international trials
Non−Industry−sponsored international trials
0
10
40
100
200
300
450
Number of clinical trials per 1 million inhabitants
