# Supplementary material for: Differential Globalization of Industry- and Non-Industry–Sponsored Clinical Trials
Source: PLoS One. 2015 Dec 14;10(12):e0145122. doi: 10.1371/journal.pone.0145122 (PMC4681996; doi:10.1371/journal.pone.0145122)
Supplement: S1 Table — (PDF) [file pone.0145122.s008.pdf]

Table S1: Summary of the number of registered trials initiated in 2006-2013 per million inhabitants per geographical region

Region	Median	Minimum	Maximum
Africa	2·08	0·05	25·92
South America	6·84	1·26	41·51
Oceania	3·65	1·40	180·20
North America	9·99	1·00	253·60
Western Europe	166·59	33·66	645·70
Eastern Europe	76·24	0·55	415·10
Asia	27·54	3·06	475·20
