# Supplementary material for: Differential Globalization of Industry- and Non-Industry–Sponsored Clinical Trials
Source: PLoS One. 2015 Dec 14;10(12):e0145122. doi: 10.1371/journal.pone.0145122 (PMC4681996; doi:10.1371/journal.pone.0145122)
Supplement: S2 Table — (PDF) [file pone.0145122.s009.pdf]

Table S2: Proportion of industry-sponsored trials per year for each geographical region

Region	2006	2007	2008	2009	2010	2011	2012
Africa	60·1	54·2	55·9	50·9	42·7	47·6	39·4
South America	59·4	59·3	58·4	52·9	48·9	43·5	51·2
Oceania	73·6	75·8	77·3	76·3	74·2	79·0	76·5
North America	32·0	33·0	34·3	32·5	31·6	34·1	32·8
Western Europe	43·9	39·9	38·6	35·9	33·8	31·0	31·2
Eastern Europe	89·2	85·0	86·0	85·7	85·9	83·2	82·7
Asia	42·5	42·5	40·5	40·5	38·5	38·1	36·4
