# Supplementary material for: Differential Globalization of Industry- and Non-Industry–Sponsored Clinical Trials
Source: PLoS One. 2015 Dec 14;10(12):e0145122. doi: 10.1371/journal.pone.0145122 (PMC4681996; doi:10.1371/journal.pone.0145122)
Supplement: S3 Table — (PDF) [file pone.0145122.s010.pdf]

Table S3: Proportion of industry-sponsored trials per year for each income group

Income	2006	2007	2008	2009	2010	2011	2012
High income	33·5	33·4	33·3	31·2	30·0	30·0	29·5
Upper middle income	59·3	54·8	51·7	49·4	47·1	44·3	40·3
Low middle income	69·2	69·6	71·0	66·7	64·3	53·3	52·2
Low income	7·3	7·3	5·5	9·9	7·3	11·5	3·3
