# Supplementary material for: Differential Globalization of Industry- and Non-Industry–Sponsored Clinical Trials
Source: PLoS One. 2015 Dec 14;10(12):e0145122. doi: 10.1371/journal.pone.0145122 (PMC4681996; doi:10.1371/journal.pone.0145122)
Supplement: S4 Table — (PDF) [file pone.0145122.s011.pdf]

Table S4: Proportion of industry-sponsored trials for each country.

Country	Ratio
Argentina	0.899
Australia	0.765
Austria	0.626
Bangladesh	0.133
Belarus	0.951
Belgium	0.706
Bosnia and H.	0.913
Brazil	0.438
Bulgaria	0.970
Burkina Faso	0.219
Canada	0.447
Chile	0.811
China	0.319
Colombia	0.835
Costa Rica	0.934
Croatia	0.838
Czech Rep.	0.920
Denmark	0.387
Dominican R.	0.798
Ecuador	0.712
Egypt	0.413
Estonia	0.926
Finland	0.638
France	0.501
Georgia	0.870
Germany	0.626
Ghana	0.200
Greece	0.694
Guatemala	0.865
Hong Kong	0.715
Hungary	0.939
Iceland	0.670
India	0.597
Indonesia	0.649
Iran	0.021
Ireland	0.626
Israel	0.400
Italy	0.584
Japan	0.796
Jordan	0.733
Kenya	0.116
South Korea	0.472
Latvia	0.972
Lebanon	0.802

Country	Ratio
Lithuania	0.933
Macedonia	0.938
Malawi	0.067
Malaysia	0.797
Mali	0.147
Mexico	0.812
Moldova	0.900
Morocco	0.810
Netherlands	0.451
New Zealand	0.820
Nigeria	0.161
Norway	0.382
Pakistan	0.330
Panama	0.819
Peru	0.841
Philippines	0.920
Poland	0.899
Portugal	0.851
Puerto Rico	0.816
Romania	0.954
Russia	0.923
Saudi Arabia	0.455
Serbia	0.918
Singapore	0.647
Slovakia	0.968
Slovenia	0.641
South Africa	0.808
Spain	0.672
Sweden	0.607
Switzerland	0.450
Taiwan	0.436
Tanzania	0.106
Thailand	0.485
Tunisia	0.705
Turkey	0.635
Uganda	0.033
Ukraine	0.972
United Arab Emirates	0.831
U.K.	0.547
United States	0.336
Venezuela	0.875
Vietnam	0.512
Zambia	0.012
