# Supplementary material for: Differential Globalization of Industry- and Non-Industry–Sponsored Clinical Trials
Source: PLoS One. 2015 Dec 14;10(12):e0145122. doi: 10.1371/journal.pone.0145122 (PMC4681996; doi:10.1371/journal.pone.0145122)
Supplement: S5 Table — (PDF) [file pone.0145122.s012.pdf]

Table S5: Proportion of international clinical trials among industry-sponsored trials for each country

Country	Ratio
Argentina	0.953
Australia	0.867
Austria	0.912
Belarus	0.990
Belgium	0.871
Bosnia and H.	0.947
Brazil	0.777
Bulgaria	0.975
Canada	0.856
Chile	0.966
China	0.406
Colombia	0.965
Costa Rica	0.958
Croatia	0.978
Czech Rep.	0.957
Denmark	0.893
Dominican Rep.	0.866
Egypt	0.848
Estonia	0.990
Finland	0.887
France	0.827
Georgia	0.931
Germany	0.752
Greece	0.920
Guatemala	0.987
Hong Kong	0.967
Hungary	0.951
Iceland	0.898
India	0.621
Indonesia	0.643
Ireland	0.953
Israel	0.728
Italy	0.905
Japan	0.310
South Korea	0.603
Latvia	0.979

Country	Ratio
Lebanon	0.946
Lithuania	0.994
Macedonia	0.921
Malaysia	0.948
Mexico	0.906
Netherlands	0.867
New Zealand	0.927
Norway	0.953
Pakistan	0.825
Panama	0.890
Peru	0.978
Philippines	0.853
Poland	0.965
Portugal	0.972
Puerto Rico	0.993
Romania	0.958
Russia	0.935
Saudi Arabia	0.911
Serbia	0.954
Singapore	0.785
Slovakia	0.960
Slovenia	0.954
South Africa	0.951
Spain	0.876
Sweden	0.854
Switzerland	0.863
Taiwan	0.834
Thailand	0.904
Tunisia	0.899
Turkey	0.928
Ukraine	0.991
United Arab Emirates	0.959
United Kingdom	0.749
United States	0.333
Venezuela	0.934
Vietnam	0.750
