# Supplementary material for: Differential Globalization of Industry- and Non-Industry–Sponsored Clinical Trials
Source: PLoS One. 2015 Dec 14;10(12):e0145122. doi: 10.1371/journal.pone.0145122 (PMC4681996; doi:10.1371/journal.pone.0145122)
Supplement: S6 Table — (PDF) [file pone.0145122.s013.pdf]

Table S6: Proportion of international trials among non-industry-sponsored trials for each country

Country	Ratio
Argentina	0.482
Australia	0.399
Austria	0.213
Bangladesh	0.103
Belgium	0.259
Brazil	0.070
Burkina Faso	0.316
Canada	0.158
Chile	0.314
China	0.035
Colombia	0.250
Croatia	0.342
Bosnia and H.	0.516
Denmark	0.097
Egypt	0.080
Finland	0.170
France	0.086
Germany	0.143
Ghana	0.339
Greece	0.210
Hong Kong	0.125
Hungary	0.609
India	0.125
Indonesia	0.397
Iran	0.006
Ireland	0.375
Israel	0.054
Italy	0.157
Japan	0.103
Kenya	0.337
Czech Rep.	0.029

Country	Ratio
Malawi	0.429
Malaysia	0.321
Mali	0.241
Mexico	0.157
Netherlands	0.112
New Zealand	0.778
Norway	0.115
Pakistan	0.172
Peru	0.563
Poland	0.388
Portugal	0.558
Puerto Rico	0.863
Romania	0.571
Dominican Rep.	0.333
Saudi Arabia	0.216
Singapore	0.152
Slovenia	0.373
South Africa	0.486
Spain	0.138
Sweden	0.191
Switzerland	0.188
Taiwan	0.029
Tanzania	0.339
Thailand	0.124
Turkey	0.071
Uganda	0.327
United Kingdom	0.109
United States	0.035
Vietnam	0.300
Zambia	0.494
