# Supplementary material for: Differential Globalization of Industry- and Non-Industry–Sponsored Clinical Trials
Source: PLoS One. 2015 Dec 14;10(12):e0145122. doi: 10.1371/journal.pone.0145122 (PMC4681996; doi:10.1371/journal.pone.0145122)
Supplement: S7 Table — (PDF) [file pone.0145122.s014.pdf]

Table S7: Distribution of country trial location of industry-sponsored trial over geographical regions per year.

Region	2006	2007	2008	2009	2010	2011	2012
Africa	0.019	0.015	0.015	0.015	0.013	0.017	0.015
South America	0.045	0.045	0.043	0.043	0.039	0.044	0.041
Oceania	0.030	0.027	0.027	0.027	0.028	0.028	0.031
North America	0.227	0.237	0.232	0.227	0.222	0.230	0.227
Western Europe	0.423	0.401	0.401	0.394	0.390	0.362	0.372
Eastern Europe	0.148	0.151	0.156	0.152	0.163	0.172	0.163
Asia	0.108	0.124	0.127	0.142	0.145	0.146	0.152
