# Supplementary material for: Differential Globalization of Industry- and Non-Industry–Sponsored Clinical Trials
Source: PLoS One. 2015 Dec 14;10(12):e0145122. doi: 10.1371/journal.pone.0145122 (PMC4681996; doi:10.1371/journal.pone.0145122)
Supplement: S8 Table — (PDF) [file pone.0145122.s015.pdf]

Table S8: Distribution of country trial location of non-industry-sponsored trial over geographical regions per year.

Region	2006	2007	2008	2009	2010	2011	2012
Africa	0.021	0.019	0.015	0.020	0.021	0.021	0.024
South America	0.024	0.023	0.025	0.027	0.027	0.035	0.023
Oceania	0.014	0.011	0.010	0.009	0.010	0.007	0.009
North America	0.531	0.494	0.477	0.450	0.433	0.389	0.397
Western Europe	0.284	0.317	0.313	0.333	0.335	0.362	0.353
Eastern Europe	0.011	0.014	0.017	0.014	0.013	0.017	0.015
Asia	0.115	0.122	0.144	0.146	0.162	0.168	0.178
