# Supplementary material for: Differential Globalization of Industry- and Non-Industry–Sponsored Clinical Trials
Source: PLoS One. 2015 Dec 14;10(12):e0145122. doi: 10.1371/journal.pone.0145122 (PMC4681996; doi:10.1371/journal.pone.0145122)
Supplement: S9 Table — (PDF) [file pone.0145122.s016.pdf]

Table S9: Distribution of country trial location of industry-sponsored trial over income groups per year.

Income	2006	2007	2008	2009	2010	2011	2012
United States	0.160	0.174	0.169	0.164	0.162	0.165	0.162
High income	0.662	0.645	0.646	0.646	0.653	0.635	0.647
Upper-middle income	0.141	0.140	0.144	0.150	0.142	0.157	0.150
Lower-middle income	0.036	0.040	0.040	0.039	0.041	0.041	0.042
Low income	0.002	0.001	0.001	0.001	0.001	0.002	0.001
