# Supplementary material for: Differential Globalization of Industry- and Non-Industry–Sponsored Clinical Trials
Source: PLoS One. 2015 Dec 14;10(12):e0145122. doi: 10.1371/journal.pone.0145122 (PMC4681996; doi:10.1371/journal.pone.0145122)
Supplement: S10 Table — (PDF) [file pone.0145122.s017.pdf]

Table S10: Distribution of country trial location of non-industry-sponsored trial over income groups per year.

Income	2006	2007	2008	2009	2010	2011	2012
United States	0.464	0.429	0.414	0.395	0.374	0.333	0.334
High income	0.442	0.474	0.476	0.489	0.503	0.521	0.516
Upper-middle income	0.061	0.067	0.083	0.086	0.088	0.101	0.102
Lower-middle income	0.018	0.019	0.017	0.019	0.021	0.031	0.033
Low income	0.016	0.011	0.009	0.011	0.013	0.013	0.015
